# Transfection of insect cell in suspension for efficient baculovirus generation

**DOI:** 10.1016/j.mex.2016.04.011

**Published:** 2016-05-04

**Authors:** S. Roest, S. Kapps-Fouthier, J. Klopp, S. Rieffel, B. Gerhartz, B. Shrestha

**Affiliations:** Protein Science Group (PSG), Novartis Institute of Biomedical Research (NIBR), Center for Proteomic Chemistry (CPC), Basel, Switzerland

**Keywords:** Transfection in suspension, Baculovirus, Insect cell, Sf9, Lipofectin, Polyethyleneimine (PEI), High-throughput

## Abstract

Baculovirus (BV) mediated insect cell expression system utilizes transfection as a first step to introduce recombinant baculovirus DNA into insect cells. Many labs are still relying on the conventional liposome based transfection method in adherent culture. Here we describe a more efficient method that can replace the existing method. This method is economical and does not require any special adjustment in existing labs.

• An innovative method of transfecting insect cells in suspension using polyethyleneimine (PEI) is described here.
• The beauty of this method is minimal intermediate manipulation of culture during transfection and virus generation.
• The method significantly reduces the chances of cross contamination of viruses while handling multiple targets and constructs as well as the other microbial contamination.

## Graphical abstract

**Figure:**
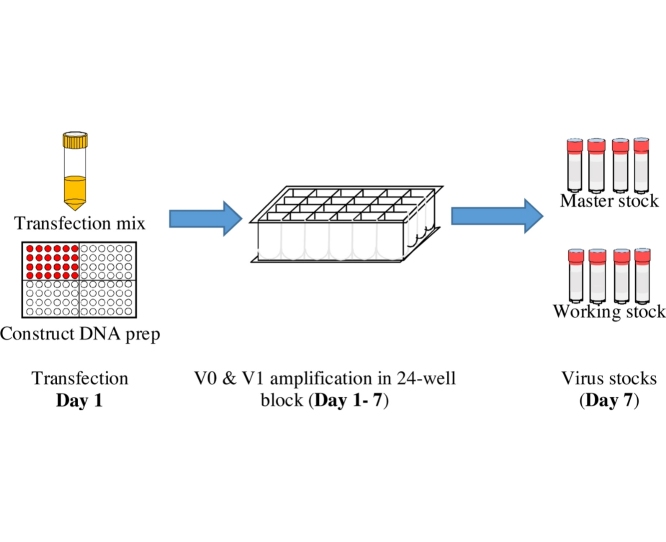

## Method details

We have described a superior method to transfect insect cells using polyethyleneimine (PEI) in suspension culture for the purpose of recombinant virus generation. The method is simple that allows transfection of multiple targets and constructs in parallel using 24-deep well block. Our lab uses FlashBac system to transfect Sf9 insect cells hence protocol is designed accordingly.

## A. Preparation of polyethylenimine (PEI)

This protocol was adapted from “Transient Expression in HEK293-EBNA1 Cells,” Chapter 12, in Expression Systems (eds. Dyson and Durocher). Scion Publishing Ltd., Oxfordshire, UK, 2007.

1. Take approximately 450 mL of Milli-Q water in a 500 mL glass beaker.
2. Add 500 mg of PEI (Cat no. 23966-2; Polysciences) in a beaker with gentle stirring.
3. Add 12 M HCl drop-wise to the solution until the pH drops <2.0.
4. Stir until the PEI is dissolved (∼2–3 h). Monitor and maintain pH <2.0 throughout. Approximately 800 μL of 12 M HCl will be required for full PEI dissolution. There may still be some small fiber-like particles that will not dissolve.
5. Add concentrated NaOH dropwise to bring solution to neutral (pH 7.0).
6. Approximately 500 μL of 10 M NaOH will be required to neutralize the PEI solution
7. Pour the solution into a 500 mL glass cylinder. Adjust the final volume to 500 mL with Milli-Q water.
8. Filter sterilize the solution through a 0.22 μm membrane filtration using vacuum filtration device.
9. Store aliquots of the desired volume at −20 °C.

## B. Routine growth and maintenance of cells

1. Sf9 cells adapted to suspension culture are maintained in Gibco^®^ Sf-900™ III SFM medium (Cat. no. 12658-027, life technologies). This serves as a master stock. Cells were passaged once the viable count reaches approximately 10 × 10^6^ cells per mL by adding fresh medium.
2. Take an aliquot of cells from the master stock and dilute to initiate 50 mL starter culture in 250 mL Erlenmeyer flask (Cat. no. 431144, Corning^®^). Recommended starting density is (0.7–0.8) × 10^6^ cells per mL.
3. Incubate diluted culture at 27 °C and 90 rpm in shaker incubator (Kuhner ISF-4-V: 50 mm rotating diameter).
4. Analyze cell count and viability using Vi-Cell™ XR cell counter (Beckman Coulter) or Neubauer’s counting chamber. It is recommended to check cell parameters every day for first few days which will give an idea on frequency of passage required. We passage cells two times a week, on day 2 (eg. Tuesday) and day 5 (eg. Friday).

*Note*: If you are new to cell culture and starting your first cell culture in suspension, please follow supplier’s instruction to revive cells from frozen stock.

## C. Maintenance of cells for transfection

In order to manage the work flow and time, Wednesday is the best time to start transfection in our set up. Hence the procedure is described accordingly. Monday is taken as a Day 1 of a week. You can adjust it to fit best for your schedule.

1. Take a small volume of cell culture from master stock and dilute to (0.7–0.8) × 10^6^ cells per mL in Sf900III medium on Day 5 of a week i.e. on Friday.
2. The following week on Day 1 (e.g. Monday), dilute cells to (0.7–0.8) × 10^6^ cells per mL in Sf900III medium.
3. On Day 2 (i.e. Tuesday) dilute further to (0.7–0.8) × 10^6^ cells per mL in fresh medium.
4. On Day 3 (i.e. Wednesday), take above culture and dilute to 0.5 × 10^6^ cells per mL. Use 1 mL of diluted culture per well of 24-deep-well block (Cat. no. 7701-5110, Whatman) for each transfection.

## D. Preparation for transfection

### Preparation of construct DNA

Methods on cloning of target DNA is beyond the scope of this communication, hence it is not explained here. The method described here follows after the gene of interest is cloned in compatible vector, produced and sequence verified.

1. Prepare isolated plasmid DNA at final concentration of 100 ng per μL.
2. Heat at 55 °C for 1h to avoid possible bacterial contamination.
3. 1.8 μL (180 ng) of it will be used for single transfection.

### Preparation of PEI-DNA complex

1. For a single transfection, take 360 μL of TC100 Medium (Cat. No. T3160, Sigma-Aldrich) and add 1.8 μL of FlashBac DNA (100 ng per μL, Oxford Expression Technologies) and 1.8 μL of PEI (1 mg per mL). For multiple transfections, prepare the master mix and aliquot before adding the DNA construct.
2. Add 1.8 μL (180 ng) of respective DNA construct cloned in FlashBac compatible vectors in it.
3. Mix gently and incubate at room temperature briefly (5–10 min). Longer incubation does not have any adverse effect.
4. Add DNA-PEI complex to cell suspension. Shake gently for first 5 h (150 rpm) then increase the speed to 450 rpm (GlasCol shaker incubator, 3 mm orbital diameter). For incubator with 25 and 50 mm orbital diameter, 250 and 200 rpm, respectively is recommended. Please optimize the speed for your incubator so that the cells do not sediment during further incubation.
5. After 5 days first generation of virus would be ready. This serves as V0 virus stock, which will be used to amplify V1 virus.

## E. V1 virus amplification

1. Use cells grown and maintained in log-phage in Sf900III medium as explained in section B. Prepare cell suspension to desired density (0.8 × 10^6^–1.0 × 10^6^ cells per mL) by adding fresh medium.
2. Add 2 mL of diluted culture in each well of 24-well block containing V0 virus.
3. Incubate the culture with 450 rpm in Glascol incubator at 27 °C.
4. Check the cell count next day (about 24 h post infection). If the cell count is high dilute it back to about 0.8–1.0 × 10^6^ cells per mL. Incubate further for 24–48 h depending on extent of infection.
5. Centrifuge the block at 4000 rpm for 10 min. Distribute the clear supernatant (V1 virus) in two halves in cryovials. Use one as a master virus stock and next as working virus stock. Both stocks are stored at 4 °C.

## F. Amplification of working virus stock

**Fig. 1:**
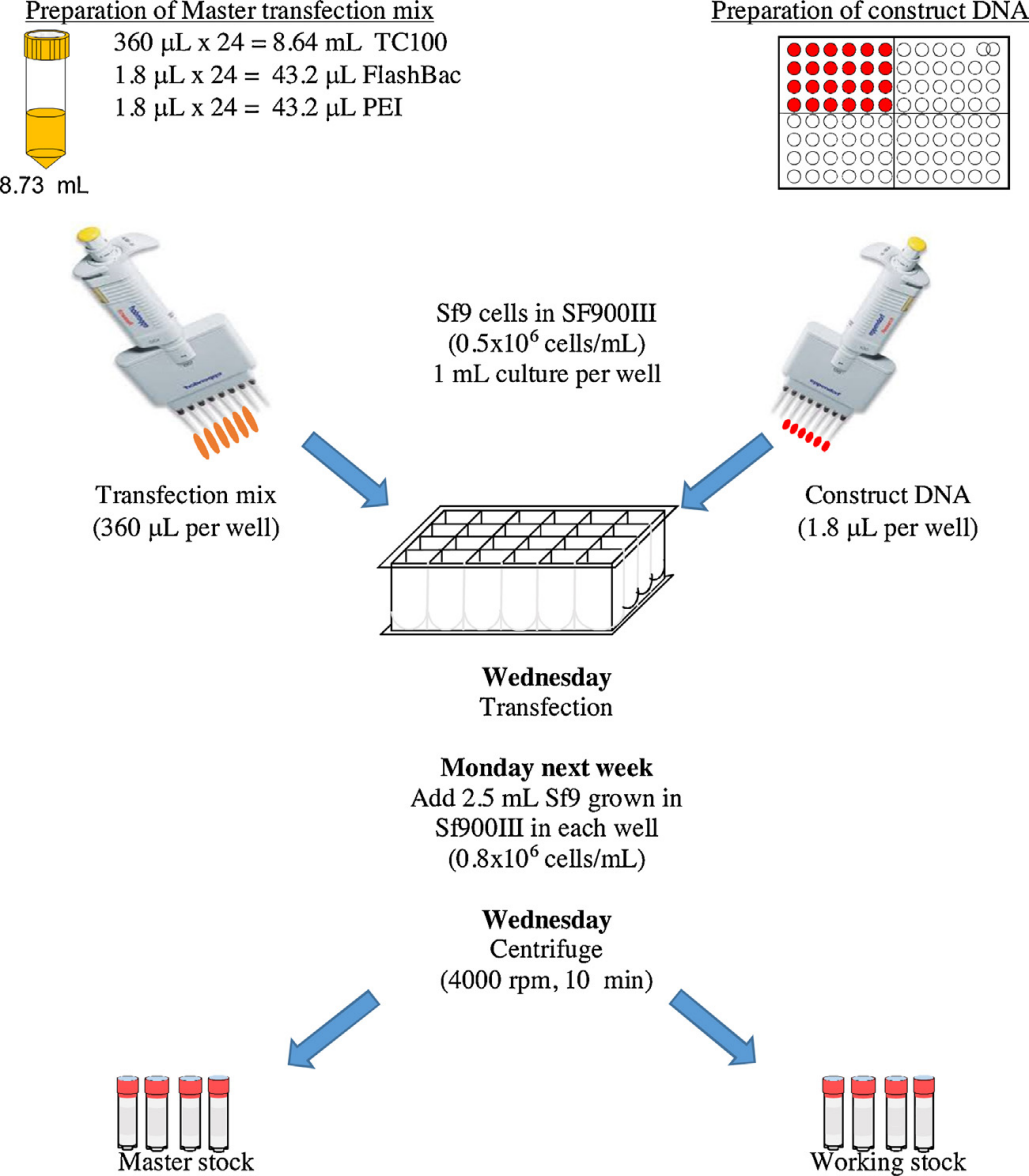
Flowchart depicting transfection in 24-deep-well block (in detail).

1. Use cells grown and maintained in log-phage in Sf900III medium as explained in section B. Prepare cell suspension to desired density (1.0 × 10^6^–1.2 × 10^6^ cells per mL) by adding fresh medium.
2. Infect with 100 μL of V1 virus for 100 mL of fresh culture.
3. Incubate at 27 °C for 48 h. Monitor in between to check the state of infection.
4. Centrifuge at 4000 rpm for 10 min. Transfer the clear supernatant into a clean sterile bottle. This will be the working stock of virus for target protein expression (Fig. 1).

## Results

### Comparison of transfection in suspension culture versus adherent culture

**Fig. 2:**
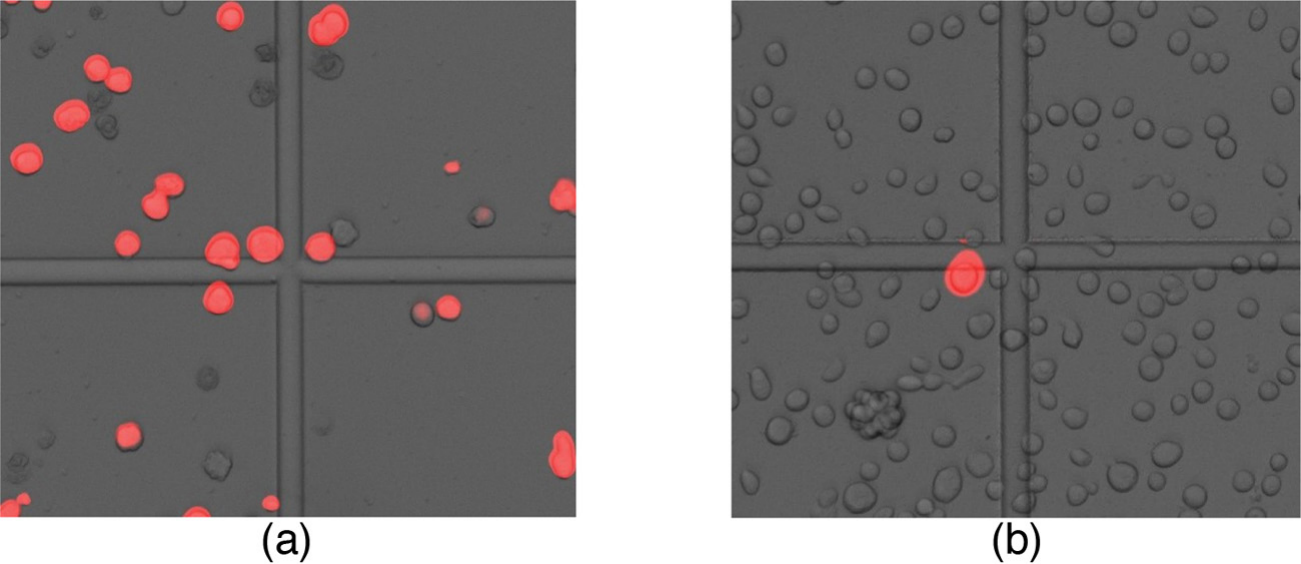
Observation of cells 48 h post infection using V0 virus generated by (a) transfection in suspension cell culture and (b) transfection in adherent cell culture of Sf9 insect cells.

Transfection of Sf9 insect cells in suspension culture was carried out as described here in method details. A procedure described by Oxford Expression Technologies (“A guide to making recombinant baculoviruses using BacPAK6 or flash BAC™ User Guide 2015,”) was followed using lipofectin as transfection agent. In order to evaluate the outcome of two procedures, comparison was done during second round of virus generation, also referred as V1 virus generation. Fig. 2 compares expression of marker protein (mCherry) 48 h post infection. In case of virus from suspension transfection (new method described here), all live cells showed the expression of mCherry and viability dropped to 59% suggesting efficient virus infection. Number of cells expressing marker mCherry was nominal and cell viability remained 97% where V0 virus was generated by monolayer transfection (conventional method).

### Effect of plasmid DNA and PEI concentration in transfection

**Fig. 3:**
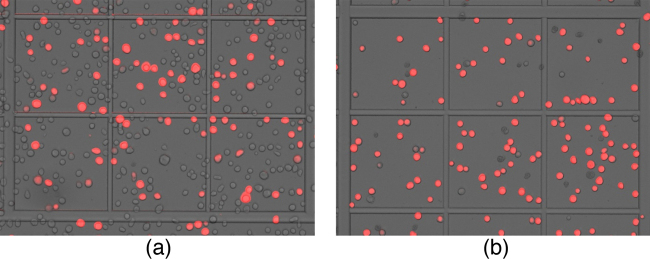
Effect of plasmid DNA concentration: (a) 180 ng and (b) 360 ng of plasmid DNA used in transfection.

**Fig. 4:**
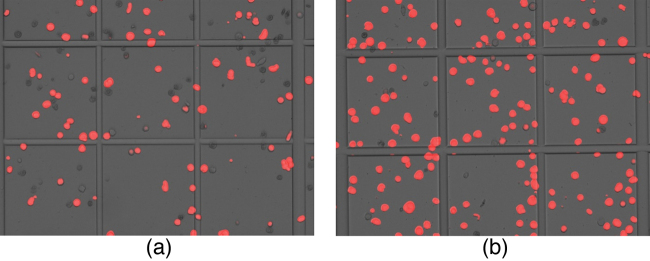
Effect of PEI concentration: (a) 1.8 μL (1.8 μg) and (b) 3.6 μL (3.6 μg) from PEI stock solution used in transfection.

Two DNA concentrations (180 ng and 360 ng) were used per transfection in order to analyze the effect. In a separate experiment, two different PEI concentrations (1.8 μg and 3.6 μg) were used. All other components of transfection mix were kept unchanged. The use of higher DNA concentration resulted in better infection (Fig. 3). On the other hand lower concentration of PEI was suitable for efficient virus production. It is recommended to optimize ratio of DNA and PEI concentration for constructs of interest when transfection efficiency appears to be sub-optimal. Less number of cells in a microscopic field (Figs. 3 and 4 ) suggests efficient infection that lead to rapid cell lysis. This has been further confirmed by cell-count, viability and increase in cell diameter (Table 1).

## Additional information

Transfection method initially developed for mammalian cell lines has been adapted for insect cells. Over the time more efficient methods were developed. Among various methods, liposome-mediated transfection (lipofection) became one of the most used methods. Transfection efficiency of about 40% could be achieved in insect cells with this method. Attempt to improve transfection efficiency in earlier work were focused for transient expression of target protein in insect cells. However, for baculovirus mediated expression system, higher efficiency of transfection is not a critical factor.

Transfection of insect cells for generation of recombinant baculoviruses is generally carried out in adherent culture using minimal medium because of the inhibitory effect of serum. This necessitates second medium exchange to serum-containing medium after the completion of transfection. The method is difficult to follow when medium or high-throughput transfection needs to be done. Transfection in suspension described here would overcome the problem. If you are using different medium than explained here the efficiency of transfection needs to be optimized. The techniques of transfection in suspension culture have been described previously. However, the aim was for the preparation of BIICs [2] or for transient expression in insect cells [3], [7].

The cationic polymer polyethylenimine (PEI) has been widely used for non-liposome based non-viral transfection *in vitro* and *in vivo*. It combines strong DNA compaction capacity with an intrinsic endosomolytic activity [5]. PEI has been successfully used to transfect different cell lines [6]. Potentiometric titration revealed that about 90% of the amines of the linear PEI homopolymer are protonated at physiological pH. Different types of cells internalize PEI/DNA complex to endolysosomal compartment. PEI combines a high membrane destabilizing potential with a high DNA condensing activity, protecting endocytosed DNA from degradation and therefore increasing the probability that intact plasmid DNA will reach the nucleus [4].

Here we combined the technique of suspension transfection with the use of PEI as a transfection reagent. There is commercial PEI commonly used for transfection in mammalian cells. However, we have prepared it in-house which would be feasible to adapt for many labs. The preparation of 50 mL PEI stock allows you to perform more than 25000 transfections. This will reduce the variation that may arise due to different batches of transfection reagent and the protocol established in an individual lab will be robust.

Useful tips:

1. Optimize transfection with different concentrations of PEI you have prepared for the best transfection outcome.
2. The concentration of plasmid DNA construct or it’s ratio to PEI may have a role in transfection efficiency.
3. If cells have grown pass the log phase, dilute and at least go for 2 passages every 24 h–36 h.
4. Optimize the shaking speed for 24-well block specifically for the type of incubator you are using. For reference, with rotating diameter of 3 mm and 50 mm, the shaking speed of 450 rpm and 200 rpm, respectively were ideal in our set up.
5. Close observation of cells after transfection is important to identify the onset of infection cycle. It is helpful to have a marker protein such as GFP or mCherry that are co-expressed with the target protein to identify the event.
6. In contrary to monolayer transfection, virus generated in first cycle will be distributed uniformly in suspension hence the expression of marker protein (if used) might not be visible. This will be much more prevalent in subsequent virus amplification for eg. V1 virus amplification.

## Figures and Tables

**Table 1:** Comparison of V1 virus amplification using virus generated from monolayer versus suspension transfection.

	Total count (×10^6^ cells/mL)	Viable count (×10^6^ cells/mL)	Viability (%)	Diameter (μm)
Cell count at the time of infection	1.66	1.59	96.3	17.49
Control (cells only)	9.84	9.51	96.7	17.49
Target 1 (V0 from monolayer)	5.31	4.93	92.9	20.53
Target 1 (V0 from suspension)	2.58	1.42	55.3	21.68
Target 2 (V0 from monolayer)	4.28	3.88	90.6	21.62
Target 2 (V0 from suspension)	2.63	1.64	62.2	22.43

Sf9 cells were used for transfection and virus generation; measurements are taken using Vi-Cell™ XR cell counter.

## References

[1] Anon (2015). A Guide to Making Recombinant Baculoviruses using BacPAK6 or Flash BAC™ User Guide Oxford Expression Technologies.

[2] Cremer H., Bechtold I., Mahnke M., Assenberg R. (2014). Efficient processes for protein expression using recombinant baculovirus particles. Anim. Cell Biotechnol..

[3] Farrell P., Iatrou K. (2004). Transfected insect cells in suspension culture rapidly yield moderate quantities of recombinant proteins in protein-free culture medium. Protein Express Purif..

[4] Klemm A.R., Young D., Lloyd J.B. (1998). Effects of polyethyleneimine on endocytosis and lysosome stability. Biochem. Pharmacol..

[5] Lungwitz U., Breunig M., Blunk T., Göpferich A. (2005). Polyethylenimine-based non-viral gene delivery systems. Eur. J. Pharm. Biopharm..

[6] Ogay I.D., Lihoradova O., a Azimova S.S., Abdukarimov a Slack J.M., Lynn D.E. (2006). Transfection of insect cell lines using polyethylenimine. Cytotechnology.

[7] Shen X., Hacker D.L., Baldi L., Wurm F.M. (2013). Virus-free transient protein production in Sf9 cells. J. Biotechnol..

